# Directionally Illuminated Autostereoscopy with Seamless Viewpoints for Multi-Viewers

**DOI:** 10.3390/mi15030403

**Published:** 2024-03-16

**Authors:** Aiqin Zhang, Xuehao Chen, Jiahui Wang, Yong He, Jianying Zhou

**Affiliations:** 1 State Key Laboratory of Optoelectronic Materials and Technology, School of Physics, Sun Yat-sen University, Guangzhou 510275, Chinastszjy@mail.sysu.edu.cn (J.Z.); 2 Guangzhou Midestereo Ltd., National University Science and Technology Park, Guangzhou 510275, China

**Keywords:** autostereoscopy, directionally illuminated design, interlaced LED backlight, seamless viewpoints, multi-viewer working mode

## Abstract

Autostereoscopy is usually perceived at finite viewpoints that result from the separated pixel array of a display system. With directionally illuminated autostereoscopy, the separation of the illumination channel from the image channel provides extra flexibility in optimizing the performance of autostereoscopy. This work demonstrates that by taking advantage of illumination freedom, seamless viewpoints in the sweet viewing region, where the ghosting does not cause significant discomfort, are realized. This realization is based on illuminating the screen with a polyline array of light emitting diodes (LEDs), and continuous viewpoints are generated through independent variation in the radiance of each individual LED column. This new method is implemented in the directionally illuminated display for both single and multiple viewers, proving its effectiveness as a valuable technique for achieving a high-quality and high-resolution autostereoscopic display with seamless viewpoints.

## 1. Introduction

Autostereoscopy with binocular parallax three-dimensional (3D) visual experience needs system design to transmit the left-/right- view images to the left-/right- eyes with minimum crosstalk [1,2,3]. The mainstream autostereoscopy approaches include the following: (1) the image splitting components (ISCs) before a liquid crystal display (LCD) [4,5,6] and (2) the directionally illuminated (DI) design behind an LCD [7,8,9,10]. The ISC technique using a parallax barrier [11,12,13], grating [14,15,16], or lenticular array [17] can be categorized as spatial multiplexing, with which the left and right view images are simultaneously loaded on the selected alternative pixels of the LCD, resulting in a resolution reduction by at least a half compared with a two-dimensional (2D) or planar display. With the ISC autostereoscopy, image information in each viewpoint comes from the corresponding pixels or subpixels behind the lenticular arrays. The interval of the viewpoints is determined by the interval of pixels (or subpixels) and the optical magnification of ISC [5,6]. Additionally, there exists a trade-off between the number of viewpoints and the resolution of single-view images. For an LCD with finite resolution, the subpixel rendering in the viewing zones is utilized [18,19]. Nevertheless, the categorization of pixels on an LCD will have to be varied for the viewer at a different viewing position, hence requiring dynamic variation in the pixel assignment if the viewer is moving around. Therefore, ISC-based high-resolution autostereoscopy is suitable for only one viewer at a time to guarantee a particular view resolution due to the specific requirement on the alignment of the pixels (or sub-pixels) with the viewer’s pupil. While multi-user autostereoscopy can be realized in principle, it is usually necessary to avoid any possible inverse vision or superimposed images. Furthermore, complete segregation of the images received with different viewers’ pupils requires that the perceived images for each viewer should be independent of each other. For this reason, the resolution perceived at a single viewpoint for spatial multiplexing is designed to be inversely proportional to the number of viewpoints. The additional requirement is that these viewpoints should be dense enough in the viewing space to provide a smooth transition between viewpoints without flickering. As an example, using an ISC to place on a 3840×2160 pixels display panel to construct 24 independent viewpoints for multi-viewers within a ±20° viewing angle, the image resolution perceived in each viewpoint will be 640×540 pixels (reduced by 1/6 horizontally and 1/4 vertically) at its best. Therefore, for an ISC-based display system, multi-viewers and a high-resolution working mode cannot be simultaneously fulfilled due to the limited resolution on the LCD panel.

For DI autostereoscopy, a full-resolution display can be achieved by alternatively loading the left- and right-view images on the LCD with a period imperceptible to the human eye [9]. The DI scheme behind the LCD is required to provide a uniform illumination on the non-self-luminous LCD panel. In addition, the DI scheme can direct the displayed images to the corresponding eye(s) of the viewer(s) through a synchronized backlight switching and eye tracking. On the one hand, as the image channel is separated from the illumination channel, it gives rise to an additional freedom to design and optimize the DI optical system via components such as volume holographic optical element, cylindrical optical elements and filed lens, etc. On the other hand, the viewpoints are basically related to the density of the backlight LEDs’ distribution. In this way, an increase in viewpoints is not at the cost of the resolution of a single viewer. Therefore, DI autostereoscopy provides an ideal approach for multi-viewers perceiving high-resolution autostereoscopy in any position in the viewing zone.

To ensure a comfortable 3D viewing experience, especially for multi-viewers, several display parameters should be simultaneously considered and optimized [20,21,22,23,24]. The experienced vision specifications, including luminance uniformity, left- and right-image crosstalk, and flicker on the screen will directly influence the 3D visual experience. The realization of luminance uniformity for a 3D display with DI becomes a bit more complex, especially in the requirement to be fulfilled in all the viewing angles (or viewpoints). The crosstalk is an exclusive parameter in 3D display which should be controlled to a minimum level. Moreover, a visible flicker is the perceived fluctuation in the brightness on the stimulus, which primarily results from deviations in a pupil from the ideal viewpoint in DI autostereoscopy. It should be emphasized that limited location accuracy eye tracking and discrete viewpoints will result in deviations in a pupil from the ideal illumination location, and the viewers will perceive a high-crosstalk image and luminance flicker, especially when viewers are moving quickly and irregularly. Our previous work [24] has applied pupil motion prediction to effectively reduce flicker for a moving viewer, and extending this motion prediction for effective pupil illumination with multi-viewer presents a technological challenge.

In this work, by taking advantage of the optimization freedom for DI autostereoscopy, we demonstrate a 3D display with seamless viewpoints for multi-viewers. We make significant improvements on the basis of our previously reported system using the techniques of densely arranged LED arrays [9,25], interlaced Fresnel lenticular arrays [22], independent LED brightness manipulation [20,21], and with intelligent eye-tracking and backlight switching [26]. Specifically, intelligent control of LED brightness is applied to achieve a seamless illumination simultaneously for multi-viewers. We show that seamless viewpoints can effectively reduce the flicker when viewers move laterally and longitudinally. Hence, to the best of our knowledge, the technique reported in this work is believed to present an ideal seamless and full-resolution autostereoscopy for multi-users for the first time.

## 2. Principle

**Figure 1:**
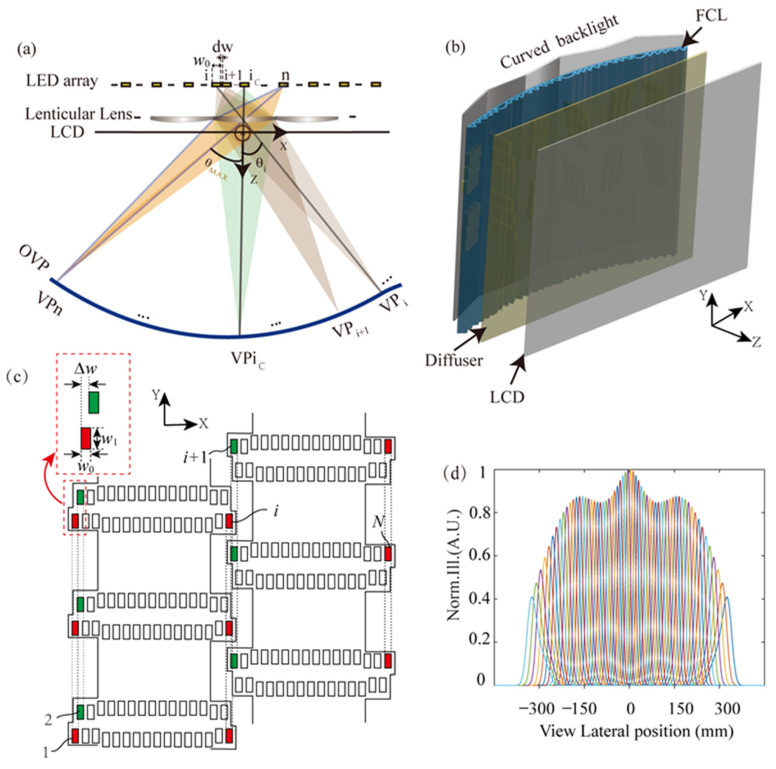
The basic principle of DI with a cylindrical lens (**a**). The schematic diagram for a stereo system with a free-form curved directional backlight module. VPi is the viewpoint of *i*th LED column, and VPic is the viewpoint at the center of the viewing zone (**b**). V-shaped interlace LED arrangement for single FL unit (**c**). The lateral normalized viewing area illuminance profiles for independently lighted on LEDs (**d**).

In a DI autostereoscopy, a planar Fresnel lens (FL) array focusing along the lateral direction is placed between an LED backlight array and the LCD, as shown in Figure 1a. With this configuration, the light rays emitting from different positioned LEDs (labeled as 1,⋯,i,i+1,⋯n) will firstly be collected by the lenticular Fresnel lens and then will pass through the near-placed LCD plane before reaching the pupil located in the optimal view plane (OVP), defined as the ideal geometrical imaging position. After carrying the loaded information on the illuminated LCD area, the light field originating from different LEDs will be focused to a series of specific areas (named the sub-viewing area) that, respectively, are located around the geometrical image positions of the LEDs. In this area, the viewer will perceive the displayed information on an LCD. For this series of discrete sub-viewing areas located in different angles *θ* in front of the LCD, they denote different viewpoints in 3D viewing experience and are labeled as $VP_{1},\cdots ,VP_{i},VP_{i+1},\cdots ,VP_{n}$.

As the LED arrays backlight is generally planarly arranged in conventional DI autostereoscopy, the OVP is distributed as a freeform curve in the top view because of the off-axis imaging of LED columns in the Fresnel lens-assisted directionally illumination system [10,27,28]. Excepting the on-axis LED column labeled as $i_{c}$, all the other columns of LEDs behind the Fresnel lens perform off-axis imaging. The farther the LED columns are from the central one, the greater their object distance will be, resulting in a smaller image distance according to the basic principles of optical imaging. Therefore, the ideal geometrical image spot position (regarded as the optimal view point) for the off-axis LED columns will be benched to the LCD screen compared with the central one, as shown in the sketch map Figure 1a, labeled by the blue line. The viewing areas in the top view around the geometric imaging position of the LED are known to have a diamond shape [10,27,28]. The diamond area is the largest in the central viewing position and it reduces to a smaller area towards the edges. The maximum view angle is $\theta_{\max}$, the size of the LED is $w_{0}\times w_{1}$, and the gap between the adjacent LEDs column is Δw. As seen in Figure 1c, the different colors (green and red) denote the LED array in different columns with a gap of Δw. If it is assumed that the LED column in the red color is the *i*th column, the LED column in the green color is the (*i* + 1)th column. For this wall-tile-like array arrangement, the dark gap between the adjacent viewing area in vision can be removed. Ignoring the aberration in the paraxial range of the FL, the viewpoints’ density is evaluated by the reciprocal of shift between the adjacent viewpoints, which is expressed by the following:

$$F_{VPD}=\frac{1}{M\cdot \Delta w}$$

where M represents the lateral magnification factor of the optical system, which is determined by the focal length of the FL and the distance between the LED and the FL.

The corresponding viewing width denoted as $w_{vp}$ at the viewpoint of a single LED, is related to the magnification of the Fresnel lens-related optical system, and can be expressed as $w_{vp}=M\cdot w_{0}$. A small-sized LED would suggest a well-defined viewing area (VA). In addition, the gap dw between adjacent LEDs will result in discrete view areas and has a relationship with Δw as $dw=-(w_{0}-\Delta w)$. Due to the existence of a finite structural boundary in each LED, the viewpoints are discretely distributed in the viewing region. The maximum allowable viewing angle $\theta_{\max}$ is mainly constrained by off-axis aberration [29,30], by the crosstalk in the viewing area, as well as by the perceived illumination uniformity on the LCD plane with the eye. To optimize the 3D view performance, a DI system with a curved LED backlight and Fresnel lenticular lens (FCL) array was developed, as shown in Figure 1b, for forming a series of sweet VAs on a flat OVP with a certain longitudinal viewing depth [13].

Considering the complexity and cost of fabrication of the freeform curved backlight configuration, a V-shaped LED arrangement behind each lens unit is adopted in this DI system, as shown in Figure 1c. The curved FCL array has a focal length of 49 mm and a lateral pitch of 32 mm for the 24-inches LCD panel. Additionally, an interlaced size of 10 mm for the adjacent FL units is applied to remove the visible optical boundaries that would exist with a conventional Fresnel lens array. In the interlaced regions of the FL, the working facets with a micron-scale size are arranged in a spatially alternating manner [14]. According to the basic geometrical imaging relationship, the curved LED backlight is set at a suitable distance so that the sweet view distance to the LCD is about 750 mm. Additionally, 62-column backlight LEDs are arranged behind each FL unit with a vertically interleaved configuration. Each LED has an illuminating width $w_{0}$ of 0.8 mm with a lateral gap Δw of 0.66 mm between the adjusted LEDs in adjusted rows. With the use of an interleaved arrangement of the LEDs, as shown in Figure 1c, the LED array is overlapped in the vertical projected direction, with an overlapping width of 0.14 mm. The numerical simulation results for the above configuration are shown Figure 1d, with the MATLAB in version of R2023. The 62 sub-viewing areas, respectively, matched up with the 62-column individual LEDs can provide a seamless viewing point distribution at the designated viewing distance. When estimating from the full width at half maximum (FWHM) of the illuminance distribution at the designated viewing distance, it is estimated that each LED illuminates in the visual plane with a distribution of FWHM at 20 mm. On the other hand, the lateral view area shift from the adjacent LED is about 10 mm, hence the seamless arrangement is transmitted from the LEDs curve to the viewing plane. The perceptible illuminance in the viewing area demonstrates a gradual decrease away from the central viewing point for equal luminance of the LEDs. Fortunately, the perceived illuminance can be optimized through the independent manipulation of the luminance of the LEDs.

**Figure 2:**
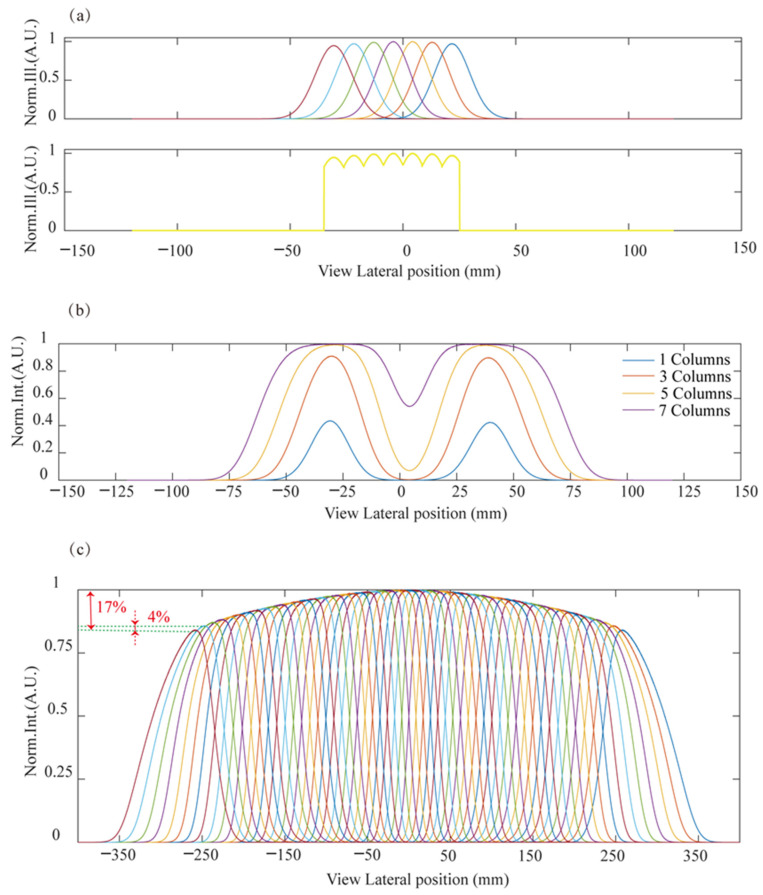
(**a**) Illuminated viewing area profiles for 7-column LEDs in the central viewing position (up) and perceived illuminance (low) at different viewing positions. (**b**) The perceived illuminance profiles around the central viewing position for the cases of simultaneously lit 1/3/5/7 column(s) of LEDs. (**c**) The combined viewing profiles by switching shifted LED arrays with a 7-column LED group. The colors used in view area profiles in (**a**,**c**) serve the purpose of distinguishing the various view areas that have overlapping parts with the surrounding view areas.

In the 3D working mode, only the backlight corresponding to the left and right pupil of the viewer needs to be switched on. However, if only one column of LEDs is on to illuminate the LCD, the viewer will experience an obvious illuminance fluctuation, as shown in Figure 2a. As the pupil is located in a different position, the perceived illuminance will vary by 88%, as calculated from Figure 2b. In fact, lighting a single column LED behind each FL unit is not sufficient to generate the uniformly illuminated viewing area required for 3D view, as shown in Figure 2a. To generate a high illuminance homogeneity, a group of LED columns are lit to provide a more uniform illuminated viewing area. It is also required that the viewing areas for the left and right eyes should not overlap, which requires that the lateral size of the viewing areas is larger than the pupil size but smaller than the interpupillary distance. As shown in Figure 2b, the lateral radiance distributions generated by 1/3/5/7 columns of LED backlight for the left and right eye were simulated. With an increasing number of lighting LED columns, the illuminance distribution becomes broadened. Thus, lighting of the group of LEDs should be optimized to provide homogeneous illuminance with minimum crosstalk. Figure 2c represents the combined viewing profile by shifting the light array composed of 7-colomn LEDs. A homogeneous illuminance can be achieved with 4% illuminance variation between adjacent sub-viewing areas and with 17% variation within the overall viewing angle. An illumination variation of 4% is obtained by the difference between the normalized illuminance at the crossing point of two adjacent view areas at the maximum viewing angle and the maximum illuminance of these two viewing areas. An illuminance uniformity of 83% is described by an illuminance variation of 17%, which is obtained by the difference between the normalized illuminance at the crossing point of two viewing areas at the maximum viewing angle and the maximum illuminance of central view area. The maximum illuminance value is normalized to 1, and the illuminance variation is also indicated in Figure 2c.

**Figure 3:**
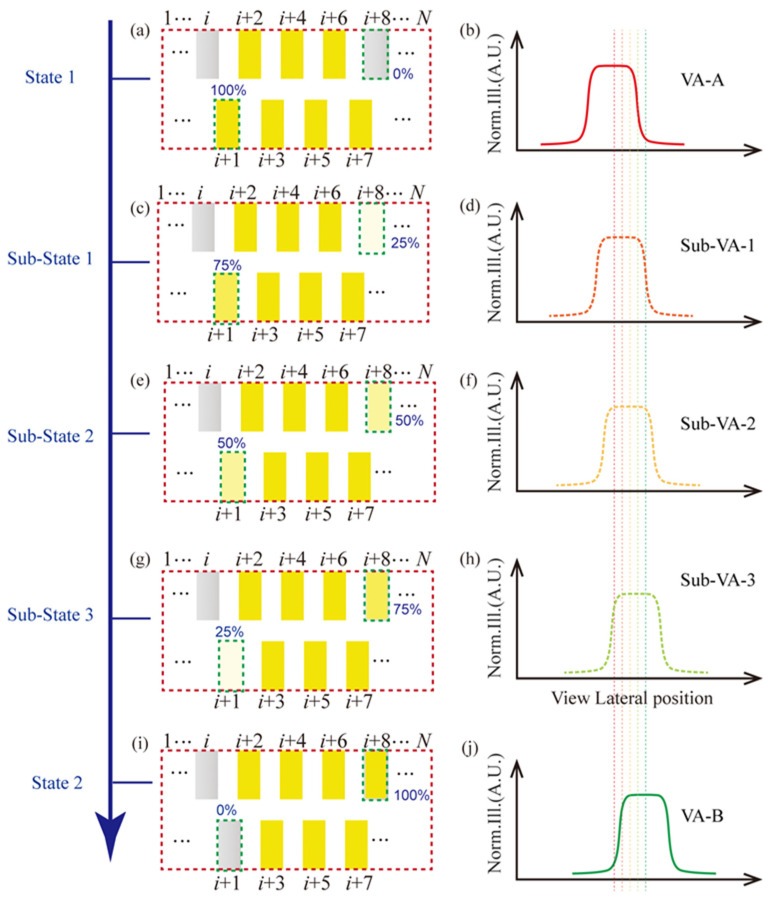
A hyperdense viewing area approach via LED weighting factor control. The left-side pictures (**a**,**c**,**e**,**g**,**i**) denote the lighting way of the LED backlight unit for forming the VA-A to VA-B, including three sub-VAs (as shown in the right-side pictures (**b**,**d**,**f**,**h**,**j**)). The yellow rectangle box denotes the state of on for an LED with maximum luminance, the gray ones denote the state of off for an LED, and the pale yellow ones denote an LED open in an altered luminance ratio, as labeled in the pictures. The dotted vertical lines that with the same color with the VA profile respectively denote the central positions of the VAs.

In principle, dense arrangements of LEDs will give rise to more closely packed viewing points. However, a higher density is observed at the cost of complex fabrication and an increased quantity of LEDs. As an alternative, the luminance from each column of LEDs can be independently controlled to further shape the viewing area distribution, as shown in Figure 3. If the luminance for any column of LEDs at the view plane is set as $I_{j}(x,y,z)$, then the perceived illuminance at any viewing area can be contributed by the 7 columns of the LEDs, which can be expressed as $I_{c}^{i}(x,y,z)=\sum_{j=i+1}^{i+7}w_{j}I_{j}(x,y,z)$, where $w_{j}$ is the weighting factor for the *j*-th LED array.

The minimum adjacent viewing area step is determined by the LED arrangement configuration, which is calculated to be approximately 10 mm without control for the LED luminous weighting factor. When the viewer changes his/her viewing position, the viewing area profile will shift correspondingly from the state 1 (as shown in Figure 3b) to state 2 (as shown in Figure 3j) by turning off the *i* + 1 column of LEDs and lighting up the *i* + 8 column of LEDs (as shown in Figure 3a,i). With the control of the weighting factor, the radiance of each LED column can be independently varied so that it can be controlled to have an infinitely small step. For example, as shown in Figure 3c–h, the radiance of the *i* + 1 column LED can be altered from 100% to 0; meanwhile for the *i* + 8, the weighting factor is assigned from 0 to 100% with 25% as a step (5 steps). As a result, the shift in adjacent viewpoints can be separated with a smaller step. The density of viewpoints can be enhanced as follows:

$$F_{VPD}=\frac{1}{M\cdot \Delta w\cdot N_{s}}$$

where $N_{s}$ indicates the number of steps. Therefore, the distance between adjacent viewpoints can be comparable or smaller than the size of the pupil, resulting in seamless viewpoints for autostereoscopy.

**Figure 4:**
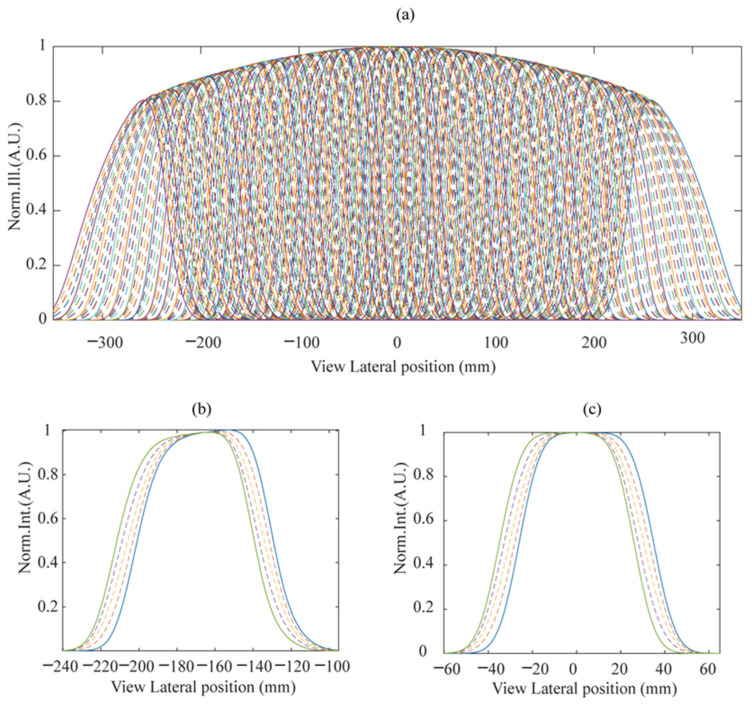
(**a**) Viewing area illuminance distribution with weighting factor manipulation for LED columns, which shows more dense viewing points than Figure 2. The enlarged-view profiles for viewpoints around the left view position of 180 mm (**b**) and central position (**c**). The colors used in the profiles serve the purpose of distinguishing the various view areas that have overlapping parts with the surrounding view areas.

Figure 4 illustrates the viewpoint distribution with LED luminous weighting factor manipulation. The solid lines represent the original viewing area achieved only by switching the LED array, while the curved lines depict the viewpoints by LED luminous weighting factor manipulation. This approach results in a triple enhancement of the viewpoint density, leading to a visual experience with higher display uniformity and reduced moving flickering. It can be anticipated that denser viewpoints can be achieved by further refining the weighting factors for each LED’s luminance.

**Figure 5:**
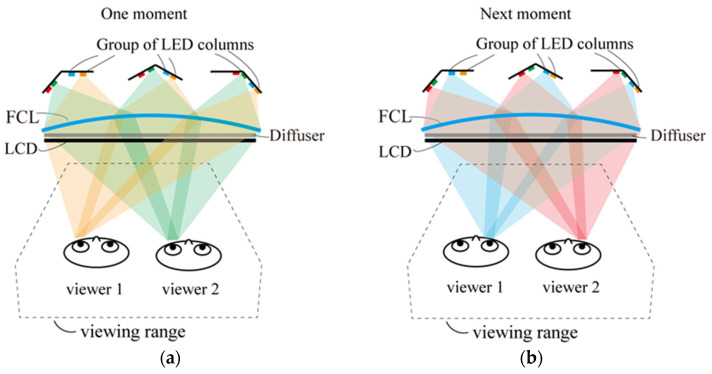
Principle diagram for the dual-viewer working mode. The light paths for (**a**) left- and (**b**) right-eye views of the dual viewers.

Figure 5 outlines the basic principle for dual viewers in stereoscopic mode. While the left-view image is being displayed on the LCD, the LED columns corresponding to the left-eye positions of the dual viewers are switched on and setup as a left-eye viewing areas for the dual viewers. The same procedure happens for the right eyes. Throughout this process, the eye-tracking system continuously monitors the viewers in real-time and provides feedback to synchronously control the LED backlight module and make the viewing area cover the eyes. The same principle is suitable for the multi-viewer working mode, as each person only needs to activate the corresponding LED columns. Even the interocular distance of the dual viewers can be different, and the position and specific lighting of the LED columns for viewers can be controlled based on the eye-tracking and backlight control system, which can ensure that both eyes of both viewers with different interocular distance are located in the optimal viewpoints.

## 3. Experiment

**Figure 6:**
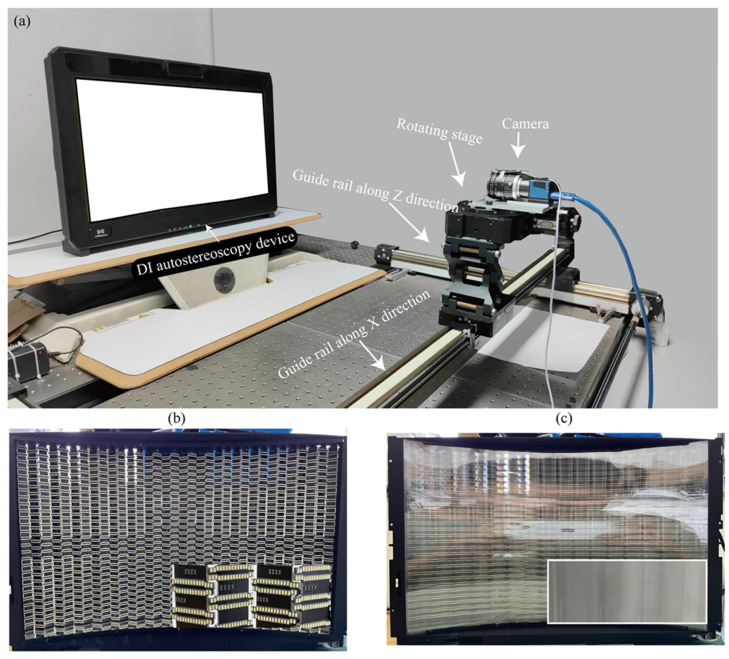
(**a**) DI autostereoscopic system and the performance test system. (**b**) The V-shaped and laterally interlaced LED backlight module. (**c**) The FL film assembled onto the LED backlight module.

The 24-inch prototype shown in Figure 6 was constructed, featuring a V-shaped interlaced LED backlight module. Optical elements such as the linear diffuser film, FL array, and LCD panel, as well as supporting structural components, a circuit module, and LCD panel and software control system, were applied. The linear diffuser film utilized in this prototype features a lateral and vertical light diffusion angle of 1° and 15°, helping to broaden the divergence angle of each backlight LED array vertically while restricting the divergence angle horizontally. This design effectively minimizes crosstalk. Figure 6b,c provides a detailed view of the critical inner structure of the prototype, with the LED backlight module and FL film placed in the system. The detailed information on the design of the interlaced FL array can be referred to in our previous work [31,32]. The FL film used in the prototype consists of 17 lens units, each with a width of 32.5 mm and a focal length of 49 mm in the experimental test section. Additionally, the lower-right insets show the part-magnified image. An auto-test system was developed to showcase the image display effects in this prototype. On the hardware side, it consists of a camera, a rotating platform, and a 2D guide rail equipped with a motor driving module. Data acquisition and motor driving are synchronized with software to control the movement of camera.

As the symmetric property with this prototype, the luminance profile at a particular viewing position was recorded. The viewpoints were chosen to be at the central viewing position and at the left-maximum viewing position, with viewing distances of 65 cm, 75 cm, and 85 cm, respectively. The weighting factor of the LED columns was controlled via modulating the driving current of the 1st and 8th column LEDs, while the columns from 2 to 7 were set to have identical luminous brightness. To test the luminance profile, the camera was placed at the central viewing position. Then, the camera was continuously moved to capture the luminance profile with a displayed white screen picture.

**Figure 7:**
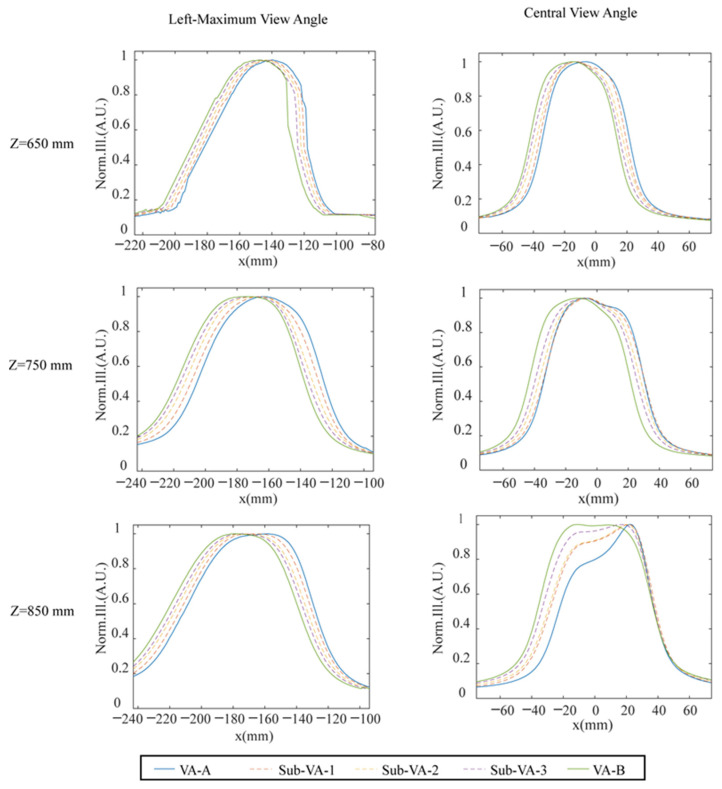
Hyperdense viewpoints for the left-maximum view angle and the central view angle at three viewing distances of 65/75/85 cm.

In the test process, the moving step for the camera was set to be 1 mm. The luminance profiles were taken at each moving position. The profile at each viewing position is represented by an average luminance from a white image on the screen, which is shown in Figure 7. The results for the three viewing distances, including 65/75/85 cm, are demonstrated for the view position around the left-maximum view angle (left side) and the central view angle (right side). The green and blue solid lines represent the profiles of the neighboring view areas obtained by switching the LED backlight module, such as from lighting on LED columns 1–7 to LED columns 2–8. Through LED brightness modulation techniques, and by opening LED columns 1–8 simultaneously but with manipulated luminosity on those LED columns, additional view areas could be achieved beyond the original adjacent view areas labeled by the green and blue solid lines, as indicated by the dotted lines. The LED’s luminance manipulation was realized by altering the current of each LED column. The luminance modulation schemes used by the LED columns to generate the extra three sub-viewing areas labeled by the dotted lines are presented in Figure 3. As seen in the central row of Figure 7, the viewpoints for the left-maximum view angle and central viewing position at the viewing distance 75 cm have similar profiles in Figure 4b,c, which suggests that the test results are in good agreement with the simulated ones. This verifies that the LED luminous control approaches can indeed increase the viewpoints for 3D display. The original viewpoint separation at 9 mm without weighting factor control can be improved to 2.5 mm with the use of LED luminous weighting factor control.

**Figure 8:**
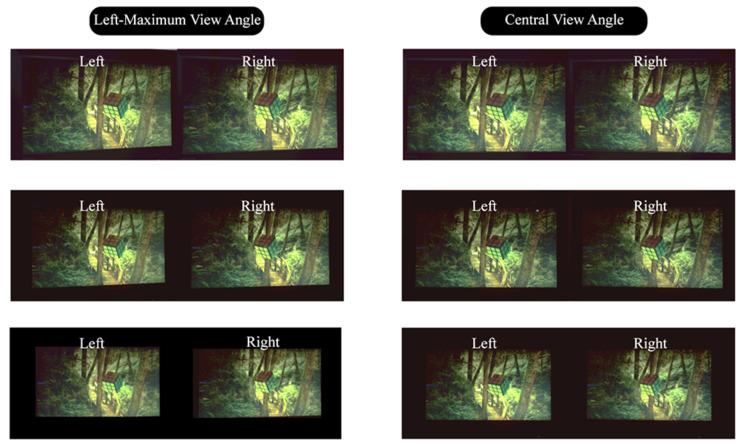
Viewing effect for a single viewer at the left-maximal view angle and the central view angle at three viewing distances of Z = 65 cm/75 cm/85 cm (first/second/third row). The left and right eyes’ visual perception show a significant disparity, which can be seen by the relative position of the tree and the small rhomboidal grid. And the disparity and brightness homogeneity are almost the same in the central viewing angle and the marginal angle.

The received images at the viewing positions were recorded by the camera placed at a series of positions that correspond to the left- and right-eye positions of a viewer at the left-maximum view angle and the central view angle from three view distances of 65 cm, 75 cm, and 85 cm, as shown in Figure 8. The left- and right-eye images received by the viewer(s) generate a 3D visual vision for the viewer(s) according to the parallaxes existing on the stereo image pairs and the human’s visual fusion effect [2]. However, the 3D visual effect cannot be directly conceived from the journal page with the naked eye. Therefore, the developed DI autostereoscopy system is necessary for the viewer. The purpose of demonstrating a side by side of the left- and right-eye received image is to illustrate the display effect of our developed protype, as the confused 3D visual quality is strongly related to the quality of the image pairs entering into the eyes. And the method of showing the stereo images for autostereoscopy adopted in this paper can be regarded as a standard method, which is widely used in the other literature [8,18]. The naked eye 3D display method studied in this paper is distinguished from the direct viewing method, uncrossed or crossed, as mentioned on page 218 in book [33], but is similar to the viewing methods with extra optical components or systems, such as those mentioned on page 220–224 of this book. The lateral distance of the two cameras is 65 mm, which is equal to the average interocular distance of humans. During the test process for each viewing position, the LEDs with luminous manipulation for both the left and right eyes are simultaneously recorded to evaluate the crosstalk. From the series of captured images, it can be seen that no significant crosstalk had occurred to produce obvious ghost imaging. The images captured for the left or right eye show no discernible variance when viewed from different perspectives and distances, which indicating a uniform 3D display quality regardless of viewing angle or distance within the designed range.

**Figure 9:**
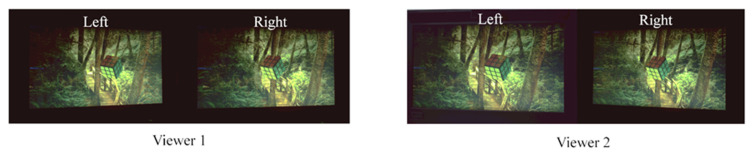
The display performance for dual viewers at a viewing distance of 750 mm. The left and right eyes’ visual perception show a significant disparity, which can be seen by the relative position of the tree and the small rhomboidal grid.

The disparity images for dual viewers simultaneously watching 3D were also recorded by the camera located, respectively, at the left- and right-eye positions. Figure 9 demonstrates the captured images for the dual-viewer vision, respectively, whilst watching 3D at the maximum-left and -right view angles, ±12°.

## 4. Discussion

**Figure 10:**
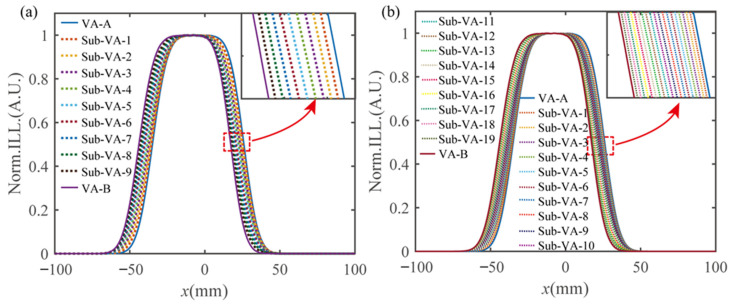
Simulation of denser view area switching. (**a**) The 9 sub-VAs and (**b**) 19 sub-VAs are generated between the original adjacent view areas VA-A and VA-B.

To further illustrate the effectiveness of LED brightness modulation techniques in increasing the visual area density, a more sophisticated LED brightness modulation ratio was employed in the simulation results, as shown in Figure 10. As the luminance of the first column of LEDs was altered from 100% to 0, the right boundary of the generated sub-viewing area (denoted by the dotted line) gradually shifted from that of the VA-A (the solid blue profile) to VA-B (the solid purple contour), as shown in the inserted image at the top right of Figure 10a,b. Inversely, the manipulation of LED luminance for the eighth column of LEDs from 0 to 100% made the left boundary of the generated sub-VA gradually shift to the left boundary of the VA-B from that of the VA-A. With a manipulation ratio of 10% and 5% on the boundary columns of the LEDs for VA-A and VA-B, 9 and 19 sub-VAs were, respectively, generated between the original adjacent VAs that were generated only by switching the LED columns. Therefore, a shift of 1 mm and 0.5 mm among the AVs can be achieved, allowing for the creation of hyperdense sweet viewpoints.

The LED luminous manipulation-based seamless viewpoints approach, as examined in this paper through simulation and experiment, has been proven to be an effective technique. It can be viewed as a software-based complementary method in the development of advanced autostereoscopy systems, as discussed in other studies such as [34,35].

## 5. Conclusions

In this work, a directionally illuminated (DI) autostereoscopic display with seamless viewpoints for multiple viewers is proposed and realized. For a V-shaped and laterally interlaced LED, the backlight module leads to a great luminance homogeneity of 83%, and the dark gaps between viewpoints are removed. By implementing LED luminous manipulation, the density of the viewpoints can be substantially improved. We demonstrate that the adjacent viewpoints can be controlled to a step of about 2 mm, which is less than the diameter of a pupil, resulting in a seamless viewing experience. This seamless scheme is demonstrated both for a single viewer and for two viewers, with the possibility to extend it to more than two viewers. We believe that the present autostereoscopy presents a practical scheme to provide a high-resolution, high-quality glasses-less 3D display, making it suitable for various autostereoscopic applications.

## Data Availability

Data are contained within the article.
